# Postbiotic *L. plantarum* RG14 improves ruminal epithelium growth, immune status and upregulates the intestinal barrier function in post-weaning lambs

**DOI:** 10.1038/s41598-019-46076-0

**Published:** 2019-07-09

**Authors:** Wan Ibrahim Izuddin, Teck Chwen Loh, Hooi Ling Foo, Anjas Asmara Samsudin, Ali Merzza Humam

**Affiliations:** 1 Department of Animal Science, Faculty of Agriculture, Universiti Putra Malaysia, 43400 UPM Serdang, Selangor Malaysia; 2 Institute of Tropical Agriculture and Food Security, Universiti Putra Malaysia, 43400 UPM Serdang, Selangor Malaysia; 3 Department of Bioprocess Technology, Faculty of Biotechnology and Biomolecular Sciences, Universiti Putra Malaysia, 43400 UPM Serdang, Selangor Malaysia; 4 Institute of Bioscience, Universiti Putra Malaysia, 43400 UPM Serdang, Selangor Malaysia; 5 Department of Animal Resources, Faculty of Agriculture, University of Baghdad, Baghdad, Iraq

**Keywords:** Animal physiology, Animal biotechnology

## Abstract

We investigate the effects of postbiotic *Lactobacillus plantarum* RG14 on gastrointestinal histology, haematology, mucosal IgA concentration, microbial population and mRNA expression related to intestinal mucosal immunity and barrier function. Twelve newly weaned lambs were randomly allocated to two treatment groups; the control group without postbiotic supplementation and postbiotic group with supplementation of 0.9% postbiotic in the diet over a 60-day trial. The improvement of rumen papillae height and width were observed in lambs fed with postbiotics. In contrast, no difference was shown in villi height of duodenum, jejunum and ileum between the two groups. Lambs received postbiotics had a lower concentration of IgA in jejunum but no difference in IgA concentration in serum and mucosal of the rumen, duodenum and ileum. In respect of haematology, postbiotics lowered leukocyte, lymphocyte, basophil, neutrophil and platelets, no significant differences in eosinophil. The increase in of IL-6 mRNA and decrease of IL-1β, IL-10, TNF mRNA were observed in the jejunum of lambs receiving postbiotics. Postbiotics also improved the integrity of the intestinal barrier by the upregulation of TJP-1, CLDN-1 and CLDN-4 mRNA. Postbiotic supplementation derived from *L. plantarum* RG14 in post-weaning lambs enhance the ruminal papillae growth, immune status and gastrointestinal health.

## Introduction

Weaning stress at the early life of production may arise from the changes in environment, social, nutrient and psychology which potentially compromise productivity, health and welfare^1^. Early weaning stressors have the potential to interrupt normal epithelial, immune, enteric nervous system which increases susceptibility to disease^2^. The enteric barrier provides a blockade from the harsh luminal environment and concurrently control the normal function of the gut such as secretion, digestion, absorption and mucosal immunity. The epithelium and lamina propria of the gastrointestinal tract are continually jeopardised to a luminal environment containing pathogens, toxins and antigens, in which the presence of gut barrier is vital to hinder an overwhelming immune activation and possibly sepsis^2^. Maintaining the barrier integrity is vital to sustain the gastrointestinal and general health of the animals. Interruption of gut mucosal integrity and barrier dysfunction result in a breach of pathogens, toxins and allergens resulting in immunological stress response and inflammation^3,4^. Nutritional strategy by supplementing with feed additive to promote balanced gut environment is one of the approaches to alleviate negative stress experienced at weaning.

In ruminants, bacterial and yeast probiotics are being used as feed additive to enhance rumen fermentation as well as to promote immune function and general health^5^. However, probiotics may contain antibiotic-resistant gene and capable of conjugating with native microbes or pathogens to transfer the gene via plasmid^6^. Alternatively, postbiotics which are the metabolites of probiotic bacteria with the absent of cells have not been investigated in ruminants particularly *in vivo* study. The mode of action of postbiotics is similar to probiotics due to the presence of metabolites of probiotic bacteria. The proposed roles of postbiotics in the gastrointestinal tract are to prevent the colonization of pathogens by improving the environment of the gut for beneficial commensal bacteria to survive and propagate. This will encourage the production of organic acids that lead to lower pH and produce more antimicrobial compounds to inhibit the proliferation of pathogenic bacteria, promote beneficial bacteria growth which modulates microbial balance, induce immune cells and immune function and prevent intestinal illness by pathogens or stress^5,7^.

Increase in population of beneficial commensal bacteria in the gut triggers the signalling of immune activation either by generating pro-inflammatory cytokines such as IL-1β, IL-12 and TNF-α or stimulate tolerance signalling by producing anti-inflammatory cytokines such as IL-10 and TGF-β^8^. In addition, production of antimicrobial peptides such as β-defensins in mucous layer in response to the presence of bacteria, viruses and fungi, are crucial to controlling the proliferation and invasion of pathogens^9^. Nevertheless, the effect of postbiotics in the modulation of immune status and gastrointestinal health particularly during key stress period in young ruminants remain unknown. Therefore, this study was conducted to assess the effect of supplementation of postbiotics from *L. plantarum* RG14 on immune status and gastrointestinal health of post-weaning lambs.

## Results

### Gastrointestinal histomorphology

Lambs supplemented with postbiotics had higher rumen papillae length and width compared to control lambs (Table 1). There was no significant difference (P > 0.05) in intestinal villi height in duodenum, jejunum and ileum between control and postbiotic groups.

**Table 1:** Gastrointestinal histology of post-weaning lambs supplemented with and without postbiotics.

Parameters	Control	Postbiotics	SEM	P-value
Rumen papillae (µm)				
Length	1394.63^a^	1913.47^b^	123.53	0.02
Width	453.20^a^	488.98^b^	7.952	0.01
Intestinal villi height (µm)				
Duodenum	402.42	452.67	18.32	0.21
Jejunum	252.28	223.52	13.76	0.35
Ileum	366.05	377.55	15.85	0.74

^a,b^Different superscripts in each row are significantly different (P < 0.05).

### Haematology

A significant higher (P < 0.05) in total leukocytes count in control group compared to postbiotic group (Table 2). In term of leukocyte types, postbiotic supplementation had significantly lower (P < 0.05) lymphocyte, basophil and neutrophil count. No difference in eosinophil count was observed between control and postbiotic group. A significantly higher (P < 0.05) in the platelet count was observed in the control group of lambs.

**Table 2:** Haematology profile of post-weaning lambs supplemented with and without postbiotics.

Blood cells (×10^9^/L)	Normal range	Control	Postbiotics	SEM	P-value
Leukocytes	4.00–12.00	7.63^a^	5.60^b^	0.52	0.04
Lymphocyte	2.00–9.00	2.33^a^	2.13^b^	0.14	0.02
Basophil	N/A	0.07^a^	0.01^b^	0.01	0.01
Eosinophil	0.00–1.00	0.10	0.08	0.02	0.61
Neutrophil	0.70–6.00	4.96^a^	3.43^b^	0.39	0.04
Platelet	250–750	1472.00^a^	971.00^b^	120.29	0.03

^a,b^Different superscripts in each row are significantly different (P < 0.05). Normal range of haematology is based on Jackson and Cockcroft^76^. N/A Not available.

### Serum and mucosal IgA

Supplementation of postbiotics did not affect IgA concentration in serum (Table 3). Level of mucosal IgA concentration in the rumen mucosal was not affected by the inclusion of postbiotics in the diet of lambs. In the small intestine mucosal, no significant differences (P > 0.05) were found for the duodenal and ileal concentration of IgA between control and postbiotic groups. However, significantly lower (P < 0.05) concentration of IgA in jejunum mucosa was observed in the postbiotic group compared to control.

**Table 3:** Serum, ruminal and intestinal mucosal IgA of post-weaning lambs supplemented with and without postbiotics.

Parameters	Control	Postbiotics	SEM	P-value
Serum (mg/ml)	1.10	0.93	0.05	0.06
Rumen (mg/g)	0.16	0.14	0.01	0.07
Duodenum (mg/g)	24.07	24.22	0.50	0.89
Jejunum (mg/g)	13.15^a^	8.96^b^	0.97	0.01
Ileum (mg/g)	2.51	2.50	0.05	0.95

^a,b^Different superscripts in each row are significantly different (P < 0.05).

### Intestinal microbial population

**Figure 1:**
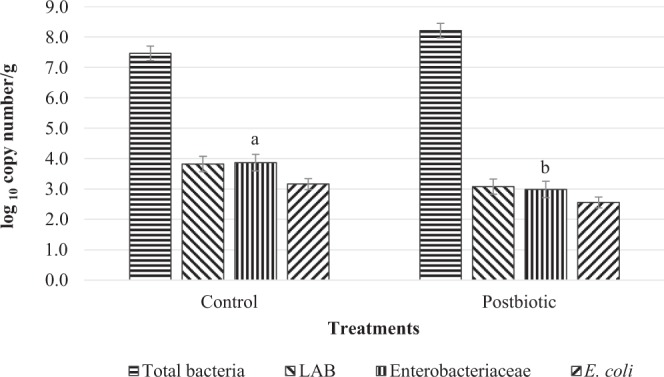
Jejunal microbial population of post-weaning lambs supplemented with and without postbiotics. LAB Lactic acid bacteria. Bar with different letter significantly differ (P < 0.05).

No significant difference (P > 0.05) was observed in the total bacteria, LAB and *E. coli* population in the jejunum between control and postbiotic groups (Fig. 1). Postbiotic supplementation had significantly lower (P < 0.05) population of Enterobacteriaceae in the jejunum of lambs.

### Intestinal mucosal immunity

**Figure 2:**
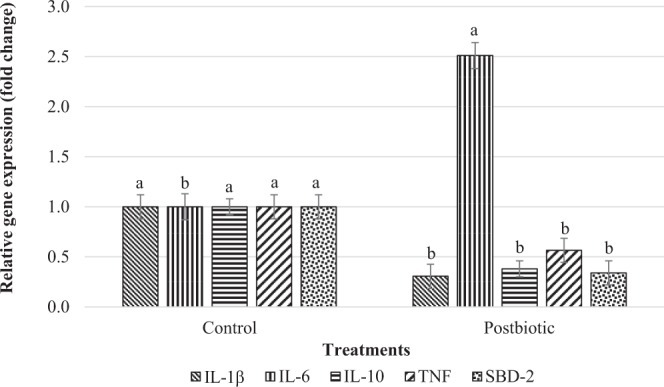
Jejunal gene expression related to mucosal immunity of post-weaning lambs supplemented with and without postbiotics. IL-1β Interleukin 1 beta; IL-6 Interleukin 6; IL-10 Interleukin 10; TNF: Tumour necrosis factor; SBD-2: Sheep beta defensin 2. Bar with different letter significantly differ (P < 0.05).

Supplementation of postbiotics downregulated the expression of pro-inflammatory cytokines, IL-1β and TNF and anti-inflammatory cytokines, IL-10 mRNA (Fig. 2). Regarding anti-microbial peptides production, SBD-2 mRNA expression was downregulated in response to postbiotic supplementation.

### Intestinal barrier function

**Figure 3:**
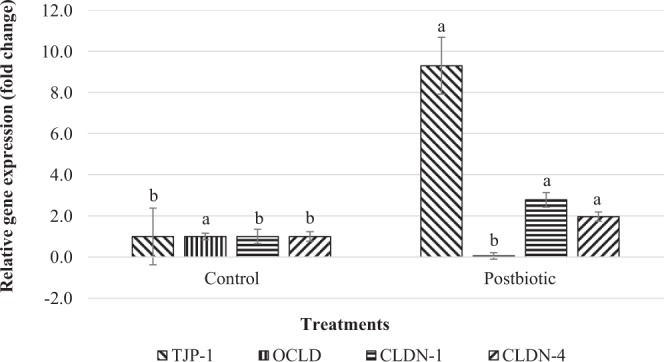
Jejunal gene expression related to intestinal barrier function of post-weaning lambs supplemented with and without postbiotics. TJP-1 Tight junction protein 1; OCLD Occludin; CLDN-1 Claudin 1; CLDN-4 Claudin 4. Bar with different letter significantly differ (P < 0.05).

Addition of postbiotics as a feed additive in the diet of post-weaning lambs upregulated the expression of TJP-1, CLDN-1 and CLDN-4 genes (Fig. 3). However, downregulation of expression of OCLD gene was observed in the postbiotic diet.

## Discussion

Lambs supplemented with postbiotics in the diet tended to have higher papillae length and width than control lambs. Rumen papillae involve three main physiological functions: (1) enlarge surface area for optimal nutrients absorption and microbial adherent to rumen wall, (2) allow products of digestion or metabolites to transfer from rumen to epithelial bed of the rumen and (3) protect the host through immune response^10,11^. The significant increase in rumen papillae may be attributed to the higher production of VFA in the rumen particularly butyric^12^. The stimulation of rumen mucosa by the VFA was also reported in the calf by Mentschel*, et al*.^13^ where the papillae length increased to 2.2 mm in the propionic acid group and 4 mm in the butyric acid group. Postbiotics have been reported to improve microbial fermentation in the caecum which produces a higher molar concentration of VFA of piglets^14,15^ and broilers^16,17^. Lane and Jesse^18^ have reported that the infusion of VFA in the rumen of lambs stimulated the metabolic and morphological development of the rumen. The higher the length and width of the papillae on rumen wall indicate the morphological development of the epithelium. Improvement of papillae length and width will increase the surface area of papillae thus increase the capacity of absorption of nutrients and attachment of microbes to rumen wall^11^. Postbiotic supplementation in the diet did not affect villi height in all parts of the small intestine in both treated and control groups. In ruminants, information on the effect of probiotics or postbiotics on post-rumen villi development is scarce. No postbiotic effect on the intestinal villi of ruminants may be due to some interaction or modification of postbiotics in the compartment of ruminant’s stomach which may not affect the hindgut morphology. In monogastric, the addition of postbiotics has been demonstrated to increase the villi height of broilers and piglets. Thu*, et al*.^14^ reported that the postbiotic inclusion in the diet of post-weaning piglets improved the villi height of duodenum but no difference in jejunal and ileal villi height. In broilers, postbiotic supplementation improved the villi height of all the segments in the small intestine^16,19^. The morphology of gut has been associated with the gut health in which enteric disease due to pathogens can lead to inflammation and compromise the development of villous-crypt structure^20^.

The finding on the higher total lymphocyte, basophil and neutrophil counts in the control group can be correlated with body inflammation in the response of infection. Neutrophils are part of the innate immune system and the central pathogen-fighting immune cells involved in phagocytosis^21,22^. They can recognize pathogens based on the unique cell-surface receptors, ingest and produce variety of toxic compounds to pathogens to eliminate diverse pathogens without the involvement of adaptive immune response^23,24^. The increase in the blood lymphocytes and basophils may indicate adaptive immune response in lambs. Lymphocytes consist of B and T cells are a major component of adaptive immune response. B cells are mediated by humoral immunity and capable of producing antibodies when stimulated by antigens. The T cells involved in cell-mediated immunity in which helper T cells secrete cytokines that assist B cells to build effective antibody response^23^. This will help to activate macrophages in recognising and phagocytosis and cytotoxic T cells precisely kill the infected cells^25^. Basophils do not engage in phagocytosis but have vital innate control of adaptive immunity that triggers the start of Th2-mediated immunity and production of immunoglobulin^26^. Elevated platelet count in the control group can be associated with occurrence of infection and inflammation in the lambs. Blood platelets involved in haemostasis, inflammation and tissue repair^27,28^. It involves in antimicrobial host defence and promotion of inflammation, and in the case of infection and inflammation platelets facilitate leukocytes in enhancing inflammatory reaction and eradicating pathogens^29^. Thus, postbiotic inclusion promotes health of the lambs and reducing the cost of immune system to respond to the antigens and pathogens.

Soon after birth, diverse bacterial communities in the gastrointestinal tract establish mutualistic and symbiotic interactions with the host^30^. The presence of beneficial microbiota defends intestinal mucosa by competing for space and nutrients with pathogens^31^. The secretory IgA and anti-microbial proteins bind to mucous layer to barricade the commensal bacteria and pathogens to the apical surface of intestinal epithelial cells^32,33^. In the current study, higher level of IgA was observed in the control group of lambs. This finding might be related to a higher number of Enterobacteriaceae in the jejunum of control group. Several probiotic and intestinal microbiota may induce B lymphocytes to produce both locally and systemic IgA by increasing the presentation of antigens by antigen presenting cell to B lymphocytes^34^. The presence of pathogens in the lumen and at intestinal epithelial barrier may have caused immune response to produce a higher level of IgA in the jejunum of lambs^33^. Payer’s patches in the intestinal mucosa are the source of IgA precursor cells which contain germinal centre that promotes antigen-specific B cells and T cells, a higher number of B cells compared to T cells by four to six times and rich in cytokines with IgA-inducing functions^35–37^. Secretory IgA in mucosal stimulates the clearance of antigens and pathogens from the intestinal lumen by barricading the entry to epithelial receptors, capture them in mucus and assist the removal by peristaltic and mucociliary activities^38^.

In the current study, a reduction of Enterobacteriaceae population was observed in the small intestine of lambs receiving postbiotics in the diet. In contrast, total bacteria, LAB and *E. coli* population in the small intestine did not differ between postbiotic and control groups. To our knowledge, the effects of probiotics or its metabolites on post-ruminal are less discovered. Postbiotics have been shown to inhibit pathogenic bacteria such as *Salmonella typhimurium*, *Eschericia coli*, *Listeria monocytogenes*, *Pediococcus acidilactici* and Vancomycin-Resistant Enterococci (VRE) *in vitro*^39,40^. In monogastric animals such as poultry and swine, the beneficial effect of postbiotics as feed additive have been reported. In broilers and layers supplemented with postbiotics, lower Enterobacteriaceae count in the digesta and faecal^16,41^. Reduction of faecal shedding of Enterobacteriaceae was also reported in post-weaning piglets fed with postbiotics in the diet^14,15^. The presence of antimicrobial metabolites such as organic acids and bacteriocins in the postbiotics might be helpful to inhibit the growth of pathogens^39,40,42^. In context of ruminants, bacteriocins and organic acids in postbiotics have to pass rumen environment and may be dissociated, metabolized or absorbed in the rumen or affected by the rumen pH regulation. However, if the organic acid such as lactic acid which is the main constituent in postbiotics is not affected in the rumen, it might help to reduce the pH and inhibit the pathogens in the post-ruminal. The ability of organic acids to pass across the cell membranes of bacteria, dissociate in the alkaline cytoplasm, thus acidify the cell interior result in inhibition of pathogenic bacteria^43^. This reduction of the population of Enterobacteriaceae in the current study may be also be associated with the stimulation of the mucosal immune system of intestine to eradicate pathogenic bacteria which would be discussed in relation with mucosal immunity. This reflects the ability of postbiotic supplementation to reduce the pathogen loads in the gastrointestinal tract.

Intestinal commensal microbiota, pathogenic bacteria and probiotics induce epithelial and immune cells at the intestinal mucosa to produce cytokines which result in the protective immune response against colonization of pathogens and regulation of intestinal barrier function^7,44,45^. In the present study, higher production of IL-6 mRNA was observed in the postbiotic group indicating the activation of humoral immune response which assists B cells in mounting potent antibody responses^46^. This can also be related to higher production and secretion of IgA by the B-cells in the mucosa of jejunum^47–49^. The cytokines involved in pro-inflammatory response such as IL-1β and IL-6 were reported to induce the production of cytokines that enhance the immune response^50^. Kareem*, et al*.^51^ reported higher expression of IL-6 mRNA in the intestinal mucosa of broilers supplemented with postbiotic RG14 and prebiotic inulin. The upregulation of IL-6 was due to higher concentrations of *Bifidobacterium* and *Lactobacillus* to prompt pro-inflammatory response^51^.

Higher expression of IL-1β and TNF in the control group may suggest the need to induce immune response due to presence of higher antigens. Similarly, a higher population of Enterobacteriaceae and secretion of IgA in the control group might be responsible for higher expression of the IL-1β and TNF^52–55^. The presence of pathogens induce the immune cells to synthesis pro-inflammatory cytokines such as IL-1β, IL-6 and TNF-α which activate B cells and helper T cells^56^. Several probiotics and intestinal commensal bacteria increase the resistance to pathogens by triggering a protective immune response which involves in the induction of pro-inflammatory cytokines and chemokines such as TNF-β, IL-8, IL-12 and IFN-γ^57^. The anti-inflammatory cytokine, IL-10 arises as a major immune-regulator at the occurrence of infection with bacteria, fungi, protozoa and viruses and helminths, improve the excessive response of Th1 and CD8^+^ T cells represented by over synthesis of IFN-γ and TNF-α^58^.

Lower expression of SBD-2 mRNA was observed in the jejunum of lambs supplemented with postbiotics. Postbiotic supplementation promoted lower population of Enterobacteriaceae in the intestine of lambs may be associated with lower SBD-2 mRNA expression. On the contrary, higher load of pathogens in the lumen or breach of pathogens within the mucous membrane or intestinal epithelial barrier will induce the response to increasing the production and secretion anti-microbial peptides into the lumen^59,60^. The β-defensins are antimicrobial peptides and have demonstrated antimicrobial activity against bacteria, fungi and enveloped viruses^9,61^ and the distribution at the mucosal surfaces is an essential first line of defence against pathogens^60,62^.

Maintaining integrity of intestinal barrier function is crucial factor in keeping healthy intestine as it helps to protect mucosal from pathogens, toxins and allergens and maintain homeostasis between immune system and gut commensal bacteria. Several agents have been reported to impact tight junction permeability and mucosal barrier functions including cytokines, growth factors, probiotics and pathogens through transcriptional regulation and post-translational modification of tight junction proteins^63,64^. The inclusion of postbiotics in the diet of post-weaning lambs increased the expression of tight junction genes. This finding supported that postbiotics which contain metabolites of probiotic bacteria having a capacity to affect the regulation of tight junction integrity and mucosal barrier function. The interactions of metabolites and bioactive molecules secreted by probiotics with intestinal immune cell receptors modulate epithelial cell function by increasing tight junction integrity and hinder its disruption^4,65^. In the current study, postbiotic supplementation increased the expression of jejunal IL-6 mRNA may be related to the increase in expression of CLDN mRNA and decrease the expression of OCLD mRNA. The TGF-β improves barrier integrity by increasing expression of CLDN-1 and CLDN-4 while IL-1β, IL-6 and TNF-α increase the permeability of intestinal cell by increasing expression of CLDN-2 and/or reducing expression of OCLD and zonula occludens (ZO)-1^63,66^. Despite the ability of tight junctions to prevent penetration of microbes, some pathogens have developed strategies as part of their pathogenesis to alter or disrupt tight junctions resulting in penetration into tissues^67^. A higher population of Enterobacteriaceae in the control group may be associated with the invasion of bacteria and linked to lower regulation of tight junction genes. Suzuki^63^ reported that enteric pathogens such as enteropathic *E. coli*, *Clostridium perfringes*, and *Vibrio cholera* are known to disrupt the intestinal barrier function by secretion of exotoxins which induce diarrhoea.

## Conclusions

The discovery of the current study suggested that supplementation of 0.9% postbiotics in the diet of post-weaning lambs increased the papillae development, lower the need to increase the production of mucosal antibodies and antimicrobial peptides, decreased the intestinal population of pathogens and reduced the cost of immune response to defence against pathogens. It can be observed that a higher generation of IL-6 but lower production IL-1β, IL-10 and TNF at the mucosal of jejunum in lambs supplemented with postbiotics. The higher production and secretion of antimicrobial peptides such as β-defensins into the lumen and regulation of intestinal barrier through tight junction proteins in lambs receiving postbiotics as can be observed in the upregulation of TJP-1, CLDN-1 and CLDN-4 further increase the health and integrity of the intestinal mucosa. Postbiotics are the potential feed additive to promote the development of rumen papillae, enhance the immune status and gastrointestinal health in the post-weaning lambs.

## Methods

### Microorganisms and maintenance

The *L. plantarum* RG14 was obtained from the Laboratory of Industrial Biotechnology, Department of Bioprocess Technology, Faculty of Biotechnology and Biomolecular Sciences, Universiti Putra Malaysia. The bacterial cultures was maintained and revived as described by Foo *et al*.^68^ and Moghadam *et al*.^69^. The bacterial cultures were maintained at −20 °C in de Man, Rogosa and Sharpe (MRS) medium (Merck, Germany) supplemented with 20% (v/v) glycerol.

### Preparation of postbiotics from *L. plantarum* RG14

The active *L. plantarum* RG14 was washed once with sterile 0.85% (w/v) NaCl (Merck, Germany) solution and adjusted to 10^9^ CFU/mL to be used as inoculum. The working cultures of *L. plantarum* RG14 was prepared by inoculating 10% (v/w) of 10^9^ CFU/mL active bacterial cell into MRS media and incubated at 30 °C for 10 hours, followed by centrifugation (Benchtop Microfuge 20 R, Beckman Coulter, Germany) at 10,000 × g, 4 °C for 15 min. The cell free supernatant (CFS) was then collected by filtration through a cellulose acetate membrane (Sartorius Minisart, 0.22 µm, Germany) as described by Loh *et al*.^70^. The CFS was stored at −20 °C until feeding trial was conducted.

### Feeding trial

The experimental protocol followed the guidelines approved by the Institutional Animal Care and Use Committee (IACUC) of the Universiti Putra Malaysia that the care and use of animals for scientific purposes are humane and ethical. The lambs used for the trial were research animals provided by the university’s research farm. The study was conducted at the Department of Animal Science Research Farm, Universiti Putra Malaysia. A total of twelve newly weaned male lambs at 112 days of age with an average body weight of 17.3 ± 0.58 kg (at the beginning of adaptation period) were randomly allocated to two treatment groups. The control group received no postbiotics and the other group received 0.9% (v/w) postbiotics in the diet. The level of postbiotics was chosen based on the previous *in vitro* study reported by *Izuddin, et al*.^71^. The lambs were individually penned and offered the isocaloric and isonitrogenous diet for 60 days including 14 days of adaptation period. The diets were formulated according to the nutritional requirements of sheep by *NRC*^72^ using FeedLIVE software (Thailand). The feed ingredients and chemical composition of the diets are presented in Table 4. The amount of grass and concentrate offered was adjusted weekly based on the four per cent of body weight of that particular week. The grass was given in the morning at 08:00 and then concentrate was added in the evening at 16:00 daily. The daily amount of individual lamb intake of grass was considered during the experimental period to ensure the concentrate given meet the grass to concentrate ratio. Fresh drinking water was continuously provided in each pen.

**Table 4:** Feed composition and nutrient content of the feed.

	Control	Postbiotic
Feed composition (%)		
Grass	30.00	30.00
Corn	40.00	40.00
Soybean	23.80	23.80
Wheat pollard	3.40	3.40
Crude palm oil	0.90	0.90
Calcium carbonate	1.70	1.70
Salt	0.40	0.40
Mineral premix^a^	0.90	0.90
Vitamin premix^b^	0.90	0.90
Postbiotic RG14	—	0.90
Chemical nutrient composition (g kg^−1^ DM)		
Crude protein	169.00	169.00
Crude fat	27.00	26.70
NDF	596.00	596.00
ADF	169.00	166.00
Lignin	146.00	146.00

^a^Mineral mix contains Co 0.6 mg, Cu 20 mg, Fe 100 mg, I 2 mg, Mn 110 mg, Se 0.2 mg, Zn 100 mg. ^b^Vitamin premix contains vitamin A 0.45 mIU/kg, vitamin B1 0.09 g/kg, vitamin B2 0.27 g/kg, vitamin B6 0.18 g/kg, vitamin B12 0.09 mg/kg, vitamin D3 0.09 mIU/kg, vitamin E 0.67 g/kg, vitamin K3 0.18 g/kg of feed, biotin 2.12 mg/kg. The diets were formulated using FeedLive International software (Thailand).

### Samples collection and analysis

Blood samples were collected from the jugular vein (5 ml into K2 EDTA BD Vacutainer® and another 5 ml into Serum BD Vacutainer® blood collection tubes) on day 58 of the feeding trial period. Blood samples in EDTA tube were subjected to haematology analysis using Abbott Cell-Dyn 3700 SL Hematology Analyzer (Abbott, IL, USA). At day 60 of the feeding trial, all the lambs were transferred to the research abattoir in the Department of Animal Science, Universiti Putra Malaysia to fast overnight in the lairage with free access to fresh drinking water. The following day, the lambs were sacrificed according to the Halal slaughtering procedure (MS1500: 2009) by severing the carotid artery and jugular vein as outlined by Malaysian Standard^73^. After slaughtering, lambs were eviscerated for the collection of rumen tissues for histology, rumen and small intestine mucosa and collection jejunal tissue and digesta. Approximately 25 cm^2^ of caudal dorsal and ventral of the rumen as described by Lesmeister*, et al*.^74^ and 5 cm long of each middle small intestine parts (duodenum, jejunum and ileum) were collected. The collected tissues were flushed with 0.01 M phosphate buffer solution, pH 7.4 (Sigma, MO, USA) and kept in 10% (v/v) buffered formalin solution for the determination of histomorphology and frozen in liquid nitrogen to keep in −80 °C freezer upon RNA extraction. The similar location of the rumen, duodenum, jejunum and ileum were flushed with 0.01 M phosphate buffer solution, pH 7.4 (Sigma, MO, USA), scrapped to collect mucosa layer and snap-frozen in liquid nitrogen before kept in −80 °C freezer upon analysis of IgA concentration.

### Serum and mucosa IgA

The serum samples were diluted with deionised water to 2000 folds. One hundred milligrams of the mucosa of rumen, duodenum, jejunum and ileum tissue samples were mixed with 1 ml of 0.01 M phosphate buffer solution, pH 7.4 (Sigma, MO, USA), sonicated in an ice-cold water bath for 30 min and stored overnight at −20 °C. Then, thawed mixtures were centrifuged at 5000 × g for 5 minutes in 4 °C. The supernatant was collected and freshly used for the analysis of IgA concentration. The concentration of IgA in serum, rumen, and small intestine tissues (duodenum, jejunum and ileum) were analysed using the Sheep IgA ELISA kit (Life Diagnostics Inc., USA) following manufacturer’s instruction. The absorbance was measured at 450 nm using microplate reader (BioTek™ ELx800™, USA). The concentration of the IgA in samples was determined by substituting the absorbance of samples into the standard curve of standards with known concentration using Gen5 Microplate Reader and Imager Software (BioTek, USA).

### Gastrointestinal histomorphology

Rumen, duodenum, jejunum and ileum tissues were cut, fixed in 10% formalin and dehydrated in an automatic tissue processor (Leica ASP 300, Japan) for 16 h. Dehydrated tissues were embedded in paraffin wax using paraffin embedding station (Leica EG 1160, Japan) and cut at 4 µm using a rotary microtome machine (Leica EG 1160, Japan). The tissues were transferred onto glass slides, heated at 58 °C for 30 minutes until dried and stained with haematoxylin and eosin. The stained tissues were examined under light microscope (Leica DM LB2, Japan) attached with a digital colour camera (Leica DFC 295, Japan). The villi height of small intestine tissue and papillae length and width for rumen tissues were measured using Leica software application suite (Leica, Japan). One tissue sample was replicated three times and for each replication, eight measurements were made (24 measurements for each sample).

### Intestinal microbial population

The isolation of microbial DNA in rumen fluid using QIAamp® Fast DNA Stool Mini Kit (Qiagen, Hilden, Germany), following the manufacturer’s protocol. The DNA concentration and purity were measured using Nanodrop 2000 spectrophotometer (Thermo Scientific, Wilmington, DE) and all biological replicate samples from each treatment (n = 6) with DNA concentration of higher than 100 ng/µL and purity was chosen for further manipulation. The populations of total bacteria, *Lactobacillus*, Enterobacteriaceae and *E. coli* were analysed by using qPCR. The targeted microbes, the sequences of the forward and reverse primers and the annealing temperature are shown in Table 5. The PCR reaction was conducted using QuantiNova™ SYBR Green PCR kit (Qiagen, Hilden, Germany) consisting a total of 20 μL PCR master mix containing 10 μL 2X SYBR Green PCR Master Mix, 1 μL forward primer, 1 μL reverse primer, 7 μL RNase-free water and 1 μL template DNA. Real-time qPCR was performed with the Bio-Rad CFX96 Real-time PCR system (Bio-Rad Laboratories, CA, USA). The primer sequence to target the specific group of bacteria was illustrated in Table 5.1. The qPCR cycling condition consisting of initial heat activation at 95 °C for 10 min, following by 40 cycles of denaturation at 95 °C for 15 s, annealing at 50 °C for *E. coli*, 55 °C for total bacteria, 58 °C for *Lactobacillus* and 60 °C for Enterobacteriaceae for 30 s and finally 30 s of extension at 72 °C. Melting curve analysis was performed at the end of the amplification cycle to confirm the specificity of amplification. Absolute quantification of microbes in the rumen fluid was performed based on the standard curve of amplification of target microbes.

**Table 5:** Primer sequence of target bacteria.

Target bacteria	Primer sequence (5′-3′)	References
Total bacteria	F - CGGCAACGAGCGCAACCC R - CCATTGTAGCACGTGTGTTAGCC	^77^
LAB	F - CATCCAGTGCAAACCTAAGAG R - GATCCGCTTGCCTTCGCA	^78^
Enterobacteriaceae	F - CATTGACGTTACCCGCAGAAGAAGC R - CTCTACGAGACTCAAGCTTGC	^79^
*E. coli*	F - GTGTGATATCTACCCGCTTCGC R - AGAACGCTTTGTGGTTAATCAGGA	^80^

F Forward, R Reverse.

### mRNA expression

Total RNA was isolated from jejunum tissues samples using RNeasy® Mini Kit (Qiagen, Hilden, Germany), following the manufacturer’s protocol. The concentration and purity of RNA were quantified by Nanodrop 2000 spectrophotometer (Thermo Scientific, Wilmington, DE) and all biological replicate samples from each treatment (n = 6) with high purity were chosen for reverse transcription. Approximately 1000 ng/µl purified RNA was converted into complementary DNA (cDNA) using Quantitect® Reverse Transcription Kit (Qiagen, Hilden, Germany) following the manufacturer’s procedure. Real-time qPCR was performed with the Bio-Rad CFX96 Real-time PCR system (Bio-Rad Laboratories, CA, USA). Table 6 shows the forward and reverse primer sequence and product size of the target and reference genes. The PCR reaction was conducted using QuantiNova™ SYBR Green PCR kit (Qiagen, Hilden, Germany) consisting a total of 20 μL PCR master mix containing 10 μL 2X SYBR Green PCR Master Mix, 1 μL forward primer, 1 μL reverse primer, 7 μL RNase-free water and 1 μL template cDNA. The qPCR cycling condition consisting of initial heat activation at 95 °C for 10 min, following by 40 cycles of denaturation at 95 °C for 15 s, annealing at 57 °C for GAPDH, CLDN-4, TNF, IL-6 and IL-10 genes, 60 °C for IL-1β, SBD-2, TJP-1, OCLD and CLDN-1 gene for 30 s and finally 30 s of extension at 72 °C. The optimum annealing temperature of target and reference genes were determined by the gradient protocol of Bio-Rad CFX96 Real-time PCR System (Bio-Rad Laboratories, CA, USA). The analysis of the melting curve was performed at the end of the amplification cycle to confirm the specificity of amplification. The relative expression of the gene was measured according to the Livak’s method of 2^−ΔΔCt^ (ΔΔCt = ΔCt treated sample − ΔCt control sample) as described by Livak and Schmittgen^75^. For the internal standard (housekeeping gene), GAPDH gene was used as to standardize the expression. The efficiency of amplification of target and housekeeping genes was determined by a 5-fold serial dilution of cDNA as a standard curve.

**Table 6:** Primer information of reference and target genes.

Target gene	Primer sequence (5′-3′)	Product size (bp)	NCBI accession number
GAPDH	F - ACCACTTTGGCATCGTGGAG R - GGGCCATCCACAGTCTTCTG	76	NM_001190390.1
IL-1β	F - CAGCCGTGCAGTCAGTAAAA R - GAAGCTCATGCAGAACACCA	137	NM_001009465.2
IL-6	F - TGCAGTCCTCAAACGAGTGGGTAA R - AGCCGCAGCTACTTCATCCGAATA	112	NM_001009392.1
IL-10	F - CCAGGATGGTGACTCGACTAGAC R - TGGCTCTGCTCTCCCAGAAC	76	NM_001009327.1
TNF	F - AACAGGCCTCTGGTTCAGACA R - CCATGAGGGCATTGGCATAC	133	NM_001024860.1
SBD-2	F - AAGCTGCCGTTGGAAGAAAG R - CCCGAAACAGGTGCCAATC	79	NM_001198545.1
TJP-1	F - CGACCAGATCCTCAGGGTAA R - AATCACCCACATCGGATTCT	161	XM_015101949.1
OCLD	F - GTTCGACCAATGCTCTCTCAG R - CAGCTCCCATTAAGGTTCCA	196	XM_015101256.1
CLDN-1	F- CACCCTTGGCATGAAGTGTA R - AGCCAATGAAGAGAGCCTGA	212	NM_001185016.1
CLDN-4	F - AAGGTGTACGACTCGCTGCT R - GACGTTGTTAGCCGTCCAG	237	NM_001185017.1

F Forward, R Reverse.

### Statistical analysis

The experiment was subjected to a completely randomized design. The differences between treatments were analysed using independent t-test procedure of SAS (Statistical Analysis System) software version 9.2 (SAS Institute, USA). Differences between treatment means were considered significant at P < 0.05.

## Data Availability

The datasets used and/or analysed during the current study are available from the corresponding author on reasonable request.
